# A novel method for in-situ extracting bio-impedance model parameters optimized for embedded hardware

**DOI:** 10.1038/s41598-023-31860-w

**Published:** 2023-03-28

**Authors:** Mitar Simić, Todd J. Freeborn, Tomislav B. Šekara, Adrian K. Stavrakis, Varun Jeoti, Goran M. Stojanović

**Affiliations:** 1 Faculty of Technical Sciences, University of Novi Sad, Trg Dositeja Obradovića 6, 21000 Novi Sad, Serbia; 2 Department of Electrical and Computer Engineering, The University of Alabama, Box 870286, Tuscaloosa, AL 35487 USA; 3 School of Electrical Engineering, University of Belgrade, Bulevar Kralja Aleksandra 73, 11120 Belgrade, Serbia

**Keywords:** Electrical and electronic engineering, Computational science

## Abstract

A novel method for embedded hardware-based parameter estimation of the Cole model of bioimpedance is developed and presented. The model parameters *R*_∞_, *R*_1_ and *C* are estimated using the derived set of equations based on measured values of real (*R*) and imaginary part (*X*) of bioimpedance, as well as the numerical approximation of the first derivative of quotient *R*/*X* with respect to angular frequency. The optimal value for parameter α is estimated using a brute force method. The estimation accuracy of the proposed method is very similar with the relevant work from the existing literature. Moreover, performance evaluation was performed using the MATLAB software installed on a laptop, as well as on the three embedded-hardware platforms (Arduino Mega2560, Raspberry Pi Pico and XIAO SAMD21). Obtained results showed that the used platforms can perform reliable bioimpedance processing with the same accuracy, while Raspberry Pi Pico is the fastest solution with the smallest energy consumption.

## Introduction

Impedance spectroscopy is a non-invasive method used in various fields, such as biology, medicine and material science^1^ to characterize the passive electrical impedance of a material or sample. It is widely applied to characterize biological tissues, where the measured impedance is often referred to as tissue bioimpedance^2^. Recently tissue bioimpedance has been utilized for respiratory monitoring during inspiratory loading, which enabled development a wearable device for detection of normal and restrictive breathing^3^. Further, combining bioimpedance with electromyography measurements have been investigated to increase the reliability of detecting muscle contraction with potential to support prosthetics and human–computer interfaces^4^. Localized bioimpedance measurements have also been utilized to monitor exercise induced changes of bicep tissues^5^. In addition to the above-mentioned applications, there are reported studies using tissue bioimpedance to non-invasively assess cardiac and respiratory activity^6^. Bioimpedance techniques have also been reported to detect of cell morphological changes in studies of ischemic rabbit liver tissue^7^ and blood glucose monitoring^8^. In recent years, significant efforts have been made to promote a personalized and patient-centric healthcare paradigm. The future healthcare will include wearable devices that will enable continuous monitoring of various parameters outside the clinics, including bioimpedance with real-time online data access^9^. Recent examples highlighting this type of system the wearable devices for knee joint health monitoring reported by Hersk et al.^10^ and Critcher and Freeborn^11^. A body composition analysis based on bioimpedance processing is important for monitoring of various chronic diseases^12^. Moreover, portable human skin surface stimulators are found to be safer if bioimpedance feedback is included to reduce the possibility of burns and injuries^13^. Further, bioimpedance based techniques are also being investigated as a sensing modality for implantable devices^14^, which opens up new health-focused applications that may not be possible using portable or wearable devices.

The traditional approaches for processing bioimpedance data can be broadly sorted into two categories: discrete processing and equivalent circuit modeling. For discrete processing, bioimpedance data (e.g. modulus/phase or real/imaginary data) collected at one or more discrete frequencies are investigated for their relationship to various health statuses of the analyzed tissue. Such an approach was used for monitoring of dialysis patients during ultrafiltration^15^, or for detection of intraneural needle placement^16^. The second approach uses equivalent electrical circuits, composed of electrical elements (such as resistors, capacitors and inductors), to represent multi-frequency bioimpedance data and then investigates the relationship of the model/component values to underlying physical and electrochemical changes in the tissue. An equivalent electrical circuit using three circuit components based on the Cole-impedance equation is widely used for various clinical applications^17^, such as body composition analysis^18^. While there are numerous studies that present the portable and wearable bioimpedance measurement devices, there are few works focused on the development of the bioimpedance processing techniques suitable for such devices^19^. A possible reason for this limited research may be that the Cole-impedance model (presented later in section "Proposed estimation method") requires the use of a fractional-order differential equation ^20,21^. Processing techniques with the Cole-impedance model have utilized complex optimizing algorithms for parameter estimation, such as non-linear least squares (NLLS)^22^, or meta-heuristic optimization algorithms^23^. A limitation of these techniques is that the accuracy, execution time and convergence are dependent on the quality of the initial values provided by the user.

Therefore, the development of bioimpedance processing techniques for parameter estimation of the Cole-impedance model that are suitable for deployment on portable embedded-hardware based devices is still an emerging topic. The aims of this paper are to formulate and evaluate a novel algorithm that is suitable for deployment on portable embedded-hardware and does not require initial-values as inputs from the user.

The rest of this work is organized as follows: The proposed estimation method is presented in section "methods and materials", as well as the characterization procedure and used hardware/software. The main experimental results are given in section "Results and discussion" with a specific focus on comparing the proposed algorithm with recent methods/hardware^19^. Further, a performance comparison of the proposed algorithm using three microcontroller-based platforms (Arduino Mega2560, Raspberry Pi Pico and XIAO SAMD21) is presented. The overall conclusions are given in section "Conclusion" with discussion about the achieved results and the practical importance of the presented method and embedded-hardware platforms.

## Methods and materials

### Proposed estimation method

The Cole-impedance model is composed of three circuit elements (two resistors and a constant phase element) with impedance represented by four parameters (*R*_*∞*_, *R*_*1*_, *C* and *α*) given by Eq. (1):

1 $$\underline{Z}\left({\omega }_{i}\right)=R\left({\omega }_{i}\right)+jX\left({\omega }_{i}\right)={R}_{\infty }+\frac{{R}_{1}}{1+{R}_{1}{C\left(j{\omega }_{i}\right)}^{\alpha }}$$

where the real part of impedance, resistance, is labelled as *R*(${\omega }_{i}$), while the imaginary part, or reactance, is labelled as *X*(${\omega }_{i}$). The constant phase element is represented by two parameters (*C* and *α)* with voltage/current characteristics modeled by a fractional-order differential equation (of order *α)*. Note that when tissue bioimpedance is measured across a limited frequency band with a fixed number (*N*) of datapoints, the frequency ($\omega )$ can be represented as a vector (${\omega }_{i})$ where *i* in as integer ranging from 1 to *N*. To simplify the presentation of the development of our method, the Cole-impedance model (Eq. (1)) is reduced to the compact form given by:

2 $$\underline{Z}\left({\omega }_{i}\right)={R}_{\infty }+\frac{{R}_{1}}{1+{R}_{1}{C\left(j{\omega }_{i}\right)}^{\alpha }}=\frac{{a}_{1}+{a}_{2}{(j{\omega }_{i})}^{\alpha }}{1+{b}_{1}{(j{\omega }_{i})}^{\alpha }}$$

where *a*_1_, *a*_2_ and *b*_1_ are coefficients with values:

3 $${a}_{1}={R}_{\infty }+{R}_{1}$$

4 $${a}_{2}={R}_{\infty }{R}_{1}C$$

5 $${b}_{1}={R}_{1}C$$

Our estimation method uses values of *R*(*ω*_*i*_) and *X*(*ω*_*i*_) obtained from the decomposition of *Z*(*ω*_*i*_) into its real and imaginary parts. All further equations assume positive values of *X*(*ω*_*i*_), which is achieved by multiplication of the actual *X*(*ω*_*i*_) with -1 prior to estimation. Both *R*(*ω*_*i*_) and *X*(*ω*_*i*_) are given with Eq. (6) and Eq. (7):

6 $$R\left({\omega }_{i}\right)=\frac{{\omega }_{i}^{\alpha }\left({a}_{1}{b}_{1}+{a}_{2}\right)\mathrm{cos}\left(\frac{\alpha \pi }{2}\right)+{\omega }_{i}^{2\alpha }{a}_{2}{b}_{1}+{a}_{1}}{1+{\omega }_{i}^{2\alpha }{b}_{1}^{2}+2{b}_{1}{\omega }_{i}^{\alpha }\mathrm{cos}\left(\frac{\alpha \pi }{2}\right)}$$

7 $$X\left({\omega }_{i}\right)=\frac{{\omega }_{i}^{\alpha }\mathrm{sin}\left(\frac{\alpha \pi }{2}\right)\left({a}_{1}{b}_{1}-{a}_{2}\right)}{1+{\omega }_{i}^{2\alpha }{b}_{1}^{2}+2{b}_{1}{\omega }_{i}^{\alpha }\mathrm{cos}\left(\frac{\alpha \pi }{2}\right)}$$

As there are four parameters to the Cole-impedance model (*a*_1_, *a*_2_, *b*_1_ and α) that are also represented in (6) and (7), an analytical solution for parameter estimation requires two additional equations. Our third equation is the first derivative with respect to *ω* of quotient *R*(*ω*)/*X*(*ω*) given by:

8 $$G^{\prime}\left( \omega \right) = \frac{\partial }{\partial \omega }\left\{ {G\left( \omega \right)} \right\} = \frac{\partial }{\partial \omega }\left\{ {\frac{R\left( \omega \right)}{{X(\omega }}} \right\} = \frac{{\alpha \left( {a_{1} - a_{2} b_{1} \omega_{i}^{2\alpha } } \right)}}{{\left( {a_{2} - a_{1} b_{1} } \right)\omega_{i}^{\alpha + 1} \sin \frac{\alpha \pi }{2}}}$$

While (8) is not directly measurable, on the discrete set of *ω*_*i*_ it can be approximated using *R*(*ω*_*i*_) and *X*(*ω*_*i*_) by a numerical procedure (central differentiation method, for example):

9 $$G^{\prime}\left( {\omega_{i} } \right) = \frac{{G\left( {\omega_{i + 1} } \right) - G\left( {\omega_{i - 1} } \right)}}{{\omega_{i + 1} - \omega_{i - 1} }} = \frac{{\frac{{R\left( {\omega_{i + 1} } \right)}}{{X\left( {\omega_{i + 1} } \right)}} - \frac{{R\left( {\omega_{i - 1} } \right)}}{{X\left( {\omega_{i - 1} } \right)}}}}{{\omega_{i + 1} - \omega_{i - 1} }}$$

In the Cole-impedance model, given by (1), the parameter α is limited to the range [0,1] and parameters *R*_*∞*_, *R*_*1*_, and *C* are limited to positive real values. Therefore, *a*_1_, *a*_2_ and *b*_1_ will be positive as well. Our estimation method solves the set of Eqs. (6)–(8) to extract parameters *a*_*1*_, *a*_*2*_, and *b*_*1*_ as a function of α, while the exact value of α is determined by a brute-force procedure to minimize the value of the defined error function.

To reduce equation length, the notation of *R*_i_, *X*_i_ and *G*_i_ for *R*(*ω*_*i*_), *X*(*ω*_*i*_) and* G*(*ω*_*i*_), respectively, was used. From (6)-(8) the values of *a*_*1*_, *a*_*2*_, and *b*_*1*_ are determined at each *ω*_*i*_ as follows:

10 $$\begin{aligned} a_{1} \left( {\omega_{i} } \right) & = - \left( {X_{i} \cdot \left( {\omega_{i}^{(2 \cdot \alpha )} \cdot \sin \left( {\left( {\pi \cdot \alpha } \right)/2} \right)^{2} \cdot \left( {R_{i}^{2} \cdot \alpha^{2} + X_{i}^{2} \cdot \alpha^{2} - G_{i}^{2} \cdot X_{i}^{2} \cdot \omega_{i}^{2} \cdot \sin \left( {\left( {\pi \cdot \alpha } \right)/2} \right)^{2} } \right)} \right)^{(1/2)} } \right. \\ & \quad + \left. {R_{i}^{2} \cdot \alpha \cdot \omega_{i}^{\alpha } \cdot \sin \left( {\left( {\pi \cdot \alpha } \right)/2} \right)^{2} + X_{i}^{2} \cdot \alpha \cdot \omega_{i}^{\alpha } \cdot \sin \left( {\left( {\pi \cdot \alpha } \right)/2} \right)^{2} - G_{i} \cdot R_{i} \cdot X_{i} \cdot \omega_{i} \cdot \omega_{i}^{\alpha } \cdot \sin \left( {\left( {\pi \cdot \alpha } \right)/2} \right)^{2} } \right)/ \\ & \quad \left( {\omega_{i}^{\alpha } \cdot \sin \left( {\left( {\pi \cdot \alpha } \right)/2} \right) \cdot \left( {X_{i} \cdot \alpha \cdot \cos \left( {\left( {\pi \cdot \alpha } \right)/2} \right) \, - R_{i} \cdot \alpha \cdot \sin \left( {\left( {\pi \cdot \alpha } \right)/2} \right) \, + G_{i} \cdot X_{i} \cdot \omega_{i} \cdot \sin \left( {\left( {\pi \cdot \alpha } \right)/2} \right)} \right)} \right) \\ \end{aligned}$$

11 $$\begin{gathered} a_{{2}} \left( {\omega_{{\text{i}}} } \right) = - \left( {R_{{\text{i}}} \cdot {\text{sin}}\left( {\left( {\pi \cdot \alpha } \right)/{2}} \right) \cdot \left( {\omega_{{\text{i}}}^{{({2} \cdot \alpha )}} \cdot {\text{sin}}\left( {\left( {\pi \cdot \alpha } \right)/{2}} \right)^{{2}} \cdot \left( {R_{{\text{i}}}^{{2}} \cdot \alpha^{{2}} + X_{{\text{i}}}^{{2}} \cdot \alpha^{{2}} - G_{{\text{i}}}^{{2}} \cdot X_{{\text{i}}}^{{2}} \cdot \omega_{{\text{i}}}^{{2}} \cdot {\text{sin}}\left( {\left( {\pi \cdot \alpha } \right)/{2}} \right)^{{2}} } \right)} \right)^{{({1}/{2})}} } \right. - \hfill \\ \left( { - X_{{\text{i}}} \cdot {\text{cos}}\left( {\left( {\pi \cdot \alpha } \right)/{2}} \right) \cdot \left( {\omega_{{\text{i}}}^{{({2} \cdot \alpha )}} \cdot {\text{sin}}\left( {\left( {\pi \cdot \alpha } \right)/{2}} \right)^{{2}} \cdot \left( {R_{{\text{i}}}^{{2}} \cdot \alpha^{{2}} + X_{{\text{i}}}^{{2}} \cdot \alpha^{{2}} - G^{{2}} \cdot X^{{2}} \cdot \omega_{{\text{i}}}^{{2}} \cdot {\text{sin}}\left( {\left( {\pi \cdot \alpha } \right)/{2}} \right)^{{2}} } \right)} \right)^{{({1}/{2})}} } \right. \hfill \\ \left( { + G_{{\text{i}}} \cdot X_{{\text{i}}}^{{2}} \cdot \omega_{{\text{i}}}^{{(\alpha \, + { 1})}} \cdot {\text{sin}}\left( {\left( {\pi \cdot \alpha } \right)/{2}} \right)^{{3}} + G_{{\text{i}}} \cdot R_{{\text{i}}} \cdot X_{{\text{i}}} \cdot \omega_{{\text{i}}}^{{(\alpha \, + { 1})}} \cdot {\text{cos}}\left( {\left( {\pi \cdot \alpha } \right)/{2}} \right) \, - G_{{\text{i}}} \cdot R_{{\text{i}}} \cdot X_{{\text{i}}} \cdot \omega_{{\text{i}}}^{{(\alpha \, + { 1})}} \cdot {\text{cos}}\left( {\left( {\pi \cdot \alpha } \right)/{2}} \right)^{{3}} } \right) \hfill \\ /\left( {\omega_{{\text{i}}}^{{({2} \cdot \alpha )}} \cdot \left( {X_{{\text{i}}} \cdot \alpha \cdot {\text{cos}}\left( {\left( {\pi \cdot \alpha } \right)/{2}} \right) \cdot {\text{sin}}\left( {\left( {\pi \cdot \alpha } \right)/{2}} \right) \, - R_{{\text{i}}} \cdot \alpha \cdot {\text{sin}}\left( {\left( {\pi \cdot \alpha } \right)/{2}} \right)^{{2}} + G_{{\text{i}}} \cdot X_{{\text{i}}} \cdot \omega_{{\text{i}}} \cdot {\text{sin}}\left( {\left( {\pi \cdot \alpha } \right)/{2}} \right)^{{2}} } \right)} \right) \hfill \\ \end{gathered}$$

12 $$\begin{gathered} b_{{1}} \left( {\omega_{{\text{i}}} } \right) = - \left( {\left( {\omega_{{\text{i}}}^{{({2} \cdot \alpha )}} \cdot {\text{sin}}\left( {\left( {\pi \cdot \alpha } \right)/{2}} \right)^{{2}} \cdot \left( {R_{{\text{i}}}^{{2}} \cdot \alpha^{{2}} + X_{{\text{i}}}^{{2}} \cdot \alpha^{{2}} - G_{{\text{i}}}^{{2}} \cdot X_{{\text{i}}}^{{2}} \cdot \omega_{{\text{i}}}^{{2}} \cdot {\text{sin}}\left( {\left( {\pi \cdot \alpha } \right)/{2}} \right)^{{2}} } \right)} \right)^{{({1}/{2})}} } \right. \hfill \\ \left. { + X_{{\text{i}}} \cdot \alpha \cdot \omega_{{\text{i}}}^{\alpha } + G_{{\text{i}}} \cdot X_{{\text{i}}} \cdot \omega_{{\text{i}}} \cdot \omega_{{\text{i}}}^{\alpha } \cdot {\text{cos}}\left( {\left( {\pi \cdot \alpha } \right)/{2}} \right) \cdot {\text{sin}}\left( {\left( {\pi \cdot \alpha } \right)/{2}} \right)} \right)/ \hfill \\ \left( {\omega_{{\text{i}}}^{{({2} \cdot \alpha )}} \cdot \left( {X_{{\text{i}}} \cdot \alpha \cdot {\text{cos}}\left( {\left( {\pi \cdot \alpha } \right)/{2}} \right) - R_{{\text{i}}} \cdot \alpha \cdot {\text{sin}}\left( {\left( {\pi \cdot \alpha } \right)/{2}} \right) + G_{{\text{i}}} \cdot X_{{\text{i}}} \cdot \omega_{{\text{i}}} \cdot {\text{sin}}\left( {\left( {\pi \cdot \alpha } \right)/{2}} \right)} \right)} \right) \hfill \\ \end{gathered}$$

The obtained arrays of *a*_*1*_ (*ω*_*i*_), *a*_*2*_(*ω*_*i*_), and *b*_*2*_(*ω*_*i*_) are averaged to calculate unique values (${\widehat{a}}_{1}$, ${\widehat{a}}_{2}$ and ${\widehat{b}}_{1}$) for a fixed value of α, which is followed by a calculation of the error function value. Next, the value of α is increased by a defined step (a step of 0.01 is used here because it provides 1% resolution) and the procedure to solve (6)–(8) with averaging is repeated.

The final value of *α* from this estimation process is selected as that specific value that minimizes the error function. In this paper we will analyze two error functions for this step. The first, referred to in this work as Method 1, estimates the real (*R*_*est*_(*ω*_*i*_)) and imaginary (*X*_*est*_(*ω*_*i*_)) impedance components using the current α and estimated ${\widehat{a}}_{1}$, ${\widehat{a}}_{2}$ and ${\widehat{b}}_{1}$ from (10)–(12) plugged into (6) and (7). Final value of α is selected such that sum of mean relative error between the estimated and measured real and imaginary part is minimal:

13 $$\underset{\alpha }{\mathrm{argmin}}\left\{\frac{1}{N}\sum_{i=1}^{N}\left|\frac{{R}_{est}(\alpha ,{\omega }_{i})-R({\omega }_{i})}{R({\omega }_{i})}\right|+\frac{1}{N}\sum_{i=1}^{N}\left|\frac{{X}_{est}(\alpha ,{\omega }_{i})-X({\omega }_{i})}{X({\omega }_{i})}\right|\right\}$$

Our second approach, referred to in this work as Method 2, selects α such that the mean absolute error between measured and estimated impedance modulus is minimal:

14 $$\underset{\alpha }{\mathrm{argmin}}\left\{\frac{1}{N}\sum_{i=1}^{N}\left|\frac{\sqrt{{R}_{est}^{2}({\alpha ,\omega }_{i})+{X}_{est}^{2}({\alpha ,\omega }_{i})}-\sqrt{{R}^{2}({\omega }_{i})+{X}^{2}({\omega }_{i})}}{\sqrt{{R}^{2}({\omega }_{i})+{X}^{2}({\omega }_{i})}}\right|\right\}$$

Finally, the estimated values of model parameters (${\widehat{R}}_{\infty }$, ${\widehat{R}}_{1}$ and $\widehat{C}$) are calculated using ${\widehat{a}}_{1}$, ${\widehat{a}}_{2}$ and ${\widehat{b}}_{1}$, and solving the set of Eqs. (3)–(5) which gives:

15 $${\widehat{R}}_{\infty }={\widehat{a}}_{2}/{\widehat{b}}_{1}$$

16 $${\widehat{R}}_{1}=\left({\widehat{a}}_{1}{\widehat{b}}_{1}-{\widehat{a}}_{2}\right) /{\widehat{b}}_{1}$$

17 $$\widehat{C}={\widehat{b}}_{1}^{2}/\left({\widehat{a}}_{1}{\widehat{b}}_{1}-{\widehat{a}}_{2}\right)$$

For better distinction from the reference values (*R*_*∞*_, *R*_*1*_, *C* and *α*) the estimated values of model parameters are labeled as (${\widehat{R}}_{\infty }$, ${\widehat{R}}_{1}$, $\widehat{C}$ and $\widehat{\alpha }$).

### Hardware platforms for estimation validation

**Figure 1:**
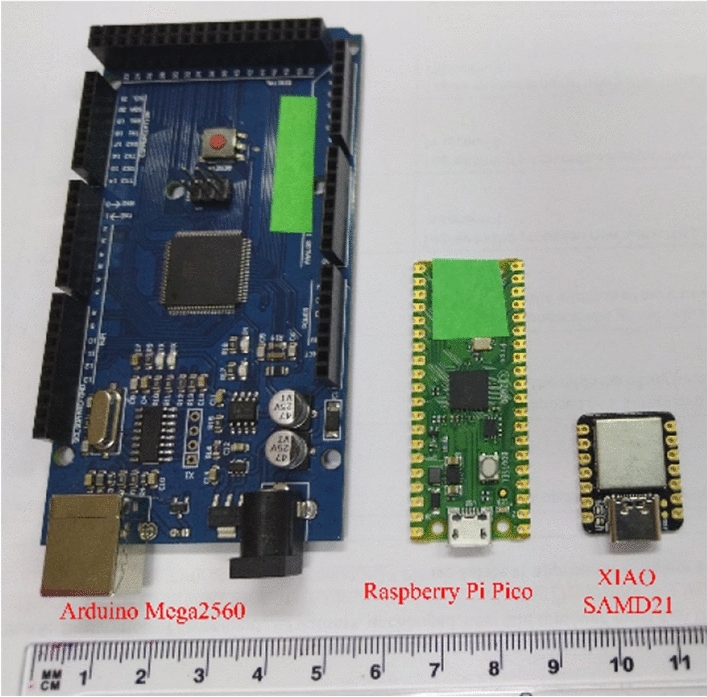
Used embedded-hardware platforms: Arduino Mega2560, Raspberry Pi Pico and XIAO SAMD21.

The proposed estimation method was developed and initially tested on a Dell G5 laptop (Core i7 9th generation) with 2.6 GHz clock speed and 16 GB of RAM. After development, the algorithm was ported to three embedded hardware platforms (Arduino Mega2560, Raspberry Pi Pico (RPi Pico) and XIAO SAMD21), shown in Fig. 1, for evaluation of parameter estimation performance.

The Arduino Mega2560 is an embedded platform that utilizes the AVR ATmega2560 8-bit microcontroller. An important feature of the ATmega2560 is the majority of instructions are executed in a single clock cycle, which can provide up to 16 Million Instructions Per Second (MIPS) at 16 MHz^24^. However, the ATmega2560 does not have built-in hardware support for floating point numbers (so floating-point operations require conversion to integer-order operations). There is 256 kB of on-board flash memory, 8 kB of RAM, and 4 kB of EEPROM storage. Overall dimensions (width × length) are: 101.52 mm × 53.3 mm with an approximate weight of 37 g. The approximate retail price of the Arduino Mega2560 platform is 48.95 USD^25^.

The Raspberry Pi Pico is an embedded platform that utilizes dual ARM Cortex-M0 + RP2040 microcontrollers which support clock speeds up to 133 MHz (with a default clock configuration of 125 MHz). The on-chip SRAM is organized into six independent banks allowing simultaneous parallel access. The Raspberry Pi Pico does not support floating point arithmetic in hardware, but it has an interpolator and integer divider peripherals with possible library optimizations. A limitation of the RP2040 is the low execution speed if the program code does not fit into the 16 kB cache. This is noticeable at application startup when nothing is cached. With the RP2040’s default settings fetching a 32-bit value requires 80 SPI clock cycles (but can be improved to 3.3 MIPS at 133 MHz^26^). In terms of storage, 2 MB of on-board flash and 256 kB of RAM are available. EEPROM is not included. The width and length are 51 mm and 21 mm. respectively, with an approximate weight of 9 g. The approximate retail price of the Raspberry Pi Pico is 4 USD^27^.

The XIAO SAMD21 is an embedded platform that utilizes the SAM D21 family of 32-bit Cortex-M0 + ARM microcontrollers. The default clock speed of XIAO SAMD21 board is 8 MHz, but can be configured to 48 MHz with the implementation of the digital frequency-locked loop. The XIAO SAMD21 does not support floating point arithmetic in hardware^28^. In terms of storage 256 kB of flash and 32 kB of RAM are available. Similar to the Raspberry Pi Pico an EEPROM is not included for XIAO SAMD21. The width and length are 23.5 mm and 17.5 mm, respectively, with an approximate weight of 9 g. The approximate retail price of the XIAO SAMD21is 5.40 USD^29^.

Comparing the specifications of the three embedded platforms, the Raspberry Pico board has the lowest price (as of accessed date from their online store), fastest clock speed, and highest amount of RAM and flash memory. However, ArduinoMega2560 board offers a greater number of available analog-to-digital (ADC) channels (but with lower resolution than RPi Pico and XIAO SAMD21) and the highest number of general-purpose input/output (GPIO) pins. Moreover, the Arduino Mega2560 has built in Electrically Erasable Programmable Read-Only Memory (EEPROM) which is useful for storing coefficients and look-up tables available after reset or powering off the device. In terms of size, the smallest area is occupied by the XIAO SAMD21 which can be very important for the wearable applications.

### Software environments

For developing and testing the estimation algorithm on the laptop computer, the MATLAB R2013b programming/computing platform was used. No additional toolboxes or toolbox functions were utilized in the development of the algorithm to support porting of the algorithm to other software tools and programming environments.

For the testing of the algorithm on the embedded hardware, the Arduino IDE 1.8.5 programming platform was used. The algorithm code for the embedded hardware used the *math* library to access the mathematical functions for manipulating floating-point numbers necessary for the algorithm. The same source code was compiled for each of the embedded platforms without any modifications.

### Synthetic datasets for performance evaluation

**Figure 2:**
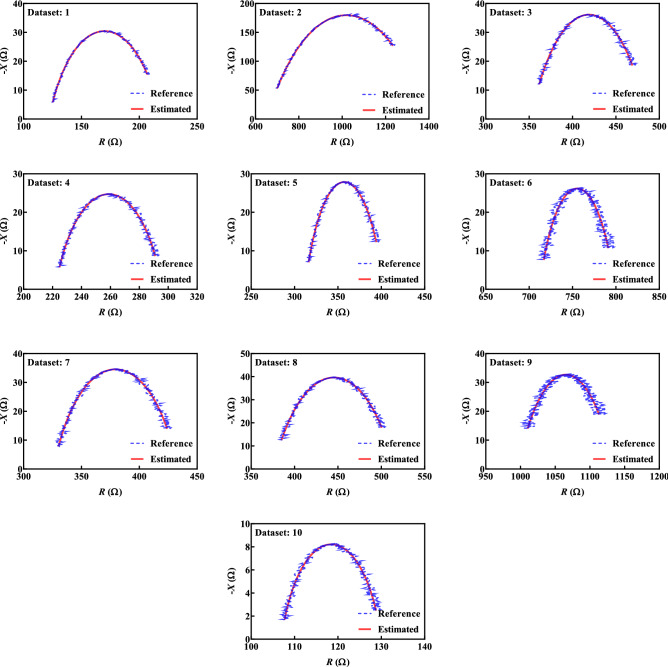
The Nyquist plots of reference and estimated values.

For the algorithm performance evaluation in terms of accuracy and execution time multiple impedance datasets were generated. Ten synthetic datasets were created using (6) and (7) with randomly chosen reference values of model parameters within the ranges: 100 Ω < *R*_*∞*_ < 1000 Ω, 10 Ω < *R*_*1*_ < 1000 Ω, 1 µF < *C* < 10 µF and 0.5 < *α* < 1.0). The specific ranges for parameters were chosen to represent values typical of bioimpedance applications. The total number of datapoints in each dataset was *N* = 256 and datapoints were logarithmically spaced in the frequency range from 3 kHz to 1 MHz (selected to represent the range and number of datapoints generated by measurements with an ImpediMed SFB7 device, widely used in bioimpedance applications). The ideal datasets had 1% random noise added to both parts of impedance (*R* and *X*) to represent bioimpedance data more accurately from tissues. For reference, these synthetic datasets are shown in Fig. 2 as solid blue lines. It is important to note that the resistance and reactance in Fig. 2 capture a wide range of values across the generation space, which ensures that the evaluation of the proposed method reflects a realistic range and not a unique or single case.

An additional synthetic dataset was created using (6) and (7) with reference values *R*_*∞*_ = 84.40 Ω, *R*_*1*_ = 39.20 Ω, *C* = 2.31 µF and *α* = 0.747 also with *N* = 256 datapoints, frequency range of 3 kHz–1 MHz, and 0.25% random noise level to replicate a test case from reference^19^ for comparison. Freeborn evaluated the performance of a least-squares parameter estimation method on both desktop/laptop and Raspberry Pi 3 platforms^19^ providing values to compare the performance of the proposed algorithm against.

To also evaluate the effect that noise has on the proposed algorithm, synthetic datasets using the reference values (*R*_*∞*_ = 84.40 Ω, *R*_*1*_ = 39.20 Ω, *C* = 2.31 µF and *α* = 0.747) were generated with: 0%, 0.25%, 1%, 5% and 10% random noise.

After that, initial dataset defined with *R*_*∞*_ = 84.40 Ω, *R*_*1*_ = 39.20 Ω, *C* = 2.31 µF, *α* = 0.747, *N* = 256 and frequency range (3 kHz–1 MHz) was divided in which lower frequency range (3 kHz–53 kHz) was used for estimation of ${\widehat{a}}_{1}$, while the part from 43 to 68 kHz was used for estimation of ${\widehat{a}}_{2}$ and ${\widehat{b}}_{1}$.

To evaluate the effect of the number of frequency points (within a fixed frequency band) on the algorithm performance, datasets using *R*_*∞*_ = 84.40 Ω, *R*_*1*_ = 39.20 Ω, *C* = 2.31 µF and *α* = 0.747 with *N* = 256, 100, 20, 10, 6, and 3 were generated using the fixed frequency band from 3 kHz to 1 MHz.

## Results and discussion

### The PC-based evaluation of the estimation method

The first evaluation of the estimation algorithm compared the execution time and parameters using the two error function definitions given by (13) and (14) when implemented in MATLAB and executed on the study laptop. These error functions are referred to as “Method 1” for (13) and “Method 2” for (14). The estimated and reference values for the synthetic 10 datasets (with 0.25% noise) are given in Table 1 for both methods. The execution time (*t*_*exe*_), as determined using the stopwatch timer embedded in MATLAB software (*tic* and *toc* functions), is also given in Table 1.

**Table 1:** Performance comparison of the proposed method in case of data with 1% of noise.

Dataset number		*t*_*exe*_	Model parameters			
1	Reference		*R*_*∞*_ = 122.00 Ω	*R*_*1*_ = 96.00 Ω	*C* = 1.90 µF	*α* = 0.72
	Method 1	0.143 s	${\widehat{R}}_{\infty }=$ 121.98 Ω	${\widehat{R}}_{1}=$ 95.97 Ω	$\widehat{C}=$ 1.90 µF	$\widehat{\alpha }=$ 0.72
	Method 2	0.151 s	${\widehat{R}}_{\infty }=$ 121.98 Ω	${\widehat{R}}_{1}=$ 95.97 Ω	$\widehat{C}=$ 1.90 µF	$\widehat{\alpha }=$ 0.72
2	Reference		*R*_*∞*_ = 652.00 Ω	*R*_*1*_ = 734.00 Ω	*C* = 1.50 µF	*α* = 0.58
	Method 1	0.097 s	${\widehat{R}}_{\infty }=$ 651.89 Ω	${\widehat{R}}_{1}=$ 733.83 Ω	$\widehat{C}=$ 1.50 µF	$\widehat{\alpha }=$ 0.58
	Method 2	0.097 s	${\widehat{R}}_{\infty }=$ 651.89 Ω	${\widehat{R}}_{1}=$ 733.83 Ω	$\widehat{C}=$ 1.50 µF	$\widehat{\alpha }=$ 0.58
3	Reference		*R*_*∞*_ = 352.00 Ω	*R*_*1*_ = 134.00 Ω	*C* = 3.20 µF	*α* = 0.63
	Method 1	0.096 s	${\widehat{R}}_{\infty }=$ 351.94 Ω	${\widehat{R}}_{1}=$ 133.97 Ω	$\widehat{C}=$ 3.20 µF	$\widehat{\alpha }=$ 0.63
	Method 2	0.101 s	${\widehat{R}}_{\infty }=$ 350.15 Ω	${\widehat{R}}_{1}=$ 139.92 Ω	$\widehat{C}=$ 3.55 µF	$\widehat{\alpha }=$ 0.62
4	Reference		*R*_*∞*_ = 222.00 Ω	*R*_*1*_ = 74.00 Ω	*C* = 1.20 µF	*α* = 0.75
	Method 1	0.142 s	${\widehat{R}}_{\infty }=$ 221.96 Ω	${\widehat{R}}_{1}=$ 73.98 Ω	$\widehat{C}=$ 1.20 µF	$\widehat{\alpha }=$ 0.75
	Method 2	0.147 s	${\widehat{R}}_{\infty }=$ 221.96 Ω	${\widehat{R}}_{1}=$ 73.98 Ω	$\widehat{C}=$ 1.20 µF	$\widehat{\alpha }=$ 0.75
5	Reference		*R*_*∞*_ = 312.00 Ω	*R*_*1*_ = 91.00 Ω	*C* = 2.02 µF	*α* = 0.70
	Method 1	0.098 s	${\widehat{R}}_{\infty }=$ 311.94 Ω	${\widehat{R}}_{1}=$ 90.98 Ω	$\widehat{C}=$ 2.02 µF	$\widehat{\alpha }=$ 0.70
	Method 2	0.099 s	${\widehat{R}}_{\infty }=$ 310.67 Ω	${\widehat{R}}_{1}=$ 95.15 Ω	$\widehat{C}=$ 2.24 µF	$\widehat{\alpha }=$ 0.69
6	Reference		*R*_*∞*_ = 712.00 Ω	*R*_*1*_ = 87.00 Ω	*C* = 2.12 µF	*α* = 0.69
	Method 1	0.097 s	${\widehat{R}}_{\infty }=$ 711.87 Ω	${\widehat{R}}_{1}=$ 86.98 Ω	$\widehat{C}=$ 2.12 µF	$\widehat{\alpha }=$ 0.69
	Method 2	0.101 s	${\widehat{R}}_{\infty }=$ 710.48 Ω	${\widehat{R}}_{1}=90$.71 Ω	$\widehat{C}=$ 2.34 µF	$\widehat{\alpha }=$ 0.68
7	Reference		*R*_*∞*_ = 326.00 Ω	*R*_*1*_ = 107.00 Ω	*C* = 1.18 µF	*α* = 0.73
	Method 1	0.146 s	${\widehat{R}}_{\infty }=$ 325.99 Ω	${\widehat{R}}_{1}=$ 106.99 Ω	$\widehat{C}=$ 1.18 µF	$\widehat{\alpha }=$ 0.73
	Method 2	0.151 s	${\widehat{R}}_{\infty }=$ 325.94 Ω	${\widehat{R}}_{1}=$ 106.97 Ω	$\widehat{C}=$ 1.18 µF	$\widehat{\alpha }=$ 0.73
8	Reference		*R*_*∞*_ = 376.00 Ω	*R*_*1*_ = 139.00 Ω	*C* = 1.98 µF	*α* = 0.66
	Method 1	0.098 s	${\widehat{R}}_{\infty }=$ 375.93 Ω	${\widehat{R}}_{1}=$ 138.97 Ω	$\widehat{C}=$ 1.98 µF	$\widehat{\alpha }=$ 0.66
	Method 2	0.100 s	${\widehat{R}}_{\infty }=$ 375.93 Ω	${\widehat{R}}_{1}=$ 138.97 Ω	$\widehat{C}=$ 1.98 µF	$\widehat{\alpha }=$ 0.66
9	Reference		*R*_*∞*_ = 996.00 Ω	*R*_*1*_ = 139.00 Ω	*C* = 6.98 µF	*α* = 0.56
	Method 1	0.097 s	${\widehat{R}}_{\infty }=$ 995.82 Ω	${\widehat{R}}_{1}=$ 138.97 Ω	$\widehat{C}=$ 6.98 µF	$\widehat{\alpha }=$ 0.56
	Method 2	0.101 s	${\widehat{R}}_{\infty }=$ 992.08 Ω	${\widehat{R}}_{1}=$ 149.15 Ω	$\widehat{C}=$ 8.58 µF	$\widehat{\alpha }=$ 0.54
10	Reference		*R*_*∞*_ = 107.00 Ω	*R*_*1*_ = 23.00 Ω	*C* = 2.28 µF	*α* = 0.79
	Method 1	0.144 s	${\widehat{R}}_{\infty }=$ 106.98 Ω	${\widehat{R}}_{1}=$ 22.99 Ω	$\widehat{C}=$ 2.28 µF	$\widehat{\alpha }=$ 0.79
	Method 2	0.154 s	${\widehat{R}}_{\infty }=$ 106.98 Ω	${\widehat{R}}_{1}=$ 22.99 Ω	$\widehat{C}=$ 2.28 µF	$\widehat{\alpha }=$ 0.79

As it can be seen from Table 1, both methods estimated values with very high accuracy, however Method 1 required smaller execution times with smaller errors. Method 1 was, in average faster for 3.5%. Therefore, Method 1 is utilized for the remainder of evaluations in this work because of its better performance. The greater execution time of Method 2 is attributed to the greater number of complex calculations in the procedure, requiring 6 multiplications of real numbers and calculation of three square roots. The Nyquist plots of the Cole impedance using the reference and estimated values with Method 1 are shown in Fig. 2. As it can be seen from Fig. 2, there is good visual agreement between the reference and estimated values for all 10 datasets.

### Comparison with reference^19^

The comparison of reference values and estimated values using our estimation method and^19^ is given in Table 2. As it can be seen from Table 2, our method estimated values with lower relative errors for all parameters except *R*_*∞*_.

**Table 2:** Performance comparison of our estimation method and CNLS method^19^.

Parameter	Reference	^19^, (MATLAB 2016a on Dell Precession 3240)	*RE* (%) Ref.^19^	This work (MATLAB 2013b on Dell G5)	*RE* (%) This work
*R*_*∞*_ (Ω)	84.40	84.56	0.193	84.62	0.255
*R*_*1*_ (Ω)	39.20	38.65	1.412	38.65	1.402
*C* (µF)	2.31	2.01	12.84	2.25	2.65
α	0.747	0.759	1.558	0.75	0.40

When compared to the CNLS-method^19^, the main advantages of the proposed method are the elimination of initial values of model parameters (to initiate the least squares search approach) and the elimination of the need for specific functions or toolboxes. For example, the initial values in^19^ are generated with 100 random sets followed by monitoring of the estimation accuracy and selection of the optimal initial values. This approach is time consuming, and it is not optimized for wearable devices based on embedded hardware. Moreover, CNLS-based method required specific the SciPy library for the Python implementation^19^.

### The impact of noise in EIS data

**Figure 3:**
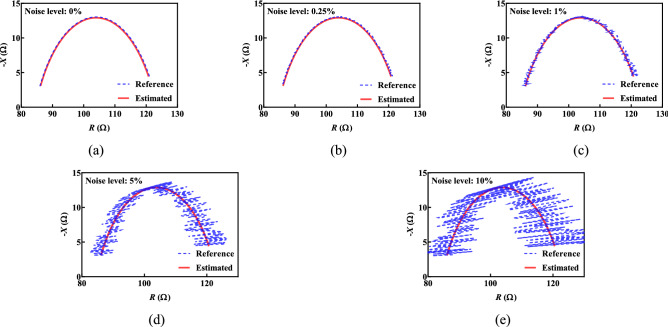
The Nyquist plots of reference and estimated values for five different noise levels: (**a**) 0%, (**b**) 0.25%, (**c**) 1%, (**d**) 5% and (**e**) 10%.

All impedance measurement devices will have different levels of accuracy (based on their underlying design) and different levels of noise (based on both design and operating environment). Since noise will always be present in a bioimpedance dataset, the performance of the estimation algorithm in terms of parameter accuracy was evaluated using the reference dataset (*R*_*∞*_ = 84.40 Ω, *R*_*1*_ = 39.20 Ω, *C* = 2.31 µF and *α* = 0.747)^19^ with five noise levels: 0%, 0.25%, 1%, 5% and 10%. Using the proposed method with these datasets, the estimated and reference impedances are shown in Fig. 3. The estimated values for the cases with 0% and 0.25% were: ${\widehat{R}}_{\infty }$=84.62 Ω, ${\widehat{R}}_{1}$=38.65 Ω, $\widehat{C}$=2.25 µF and $\widehat{\alpha }$=0.75. With increased noise to 1%, there is a very small increase in the estimation errors: ${\widehat{R}}_{\infty }$=84.60 Ω, ${\widehat{R}}_{1}$=38.64 Ω, $\widehat{C}$=2.25 µF and $\widehat{\alpha }$=0.75. Most importantly, even in cases of higher noise levels (5% and 10%), the estimated values are still close to the reference: ${\widehat{R}}_{\infty }$=84.55 Ω, ${\widehat{R}}_{1}$=38.61 Ω, $\widehat{C}$=2.25 µF and $\widehat{\alpha }$=0.75 and ${\widehat{R}}_{\infty }$=84.48 Ω, ${\widehat{R}}_{1}$=38.57 Ω, $\widehat{C}$=2.25 µF and $\widehat{\alpha }$=0.75, respectively. This supports the assumption that even with a noisy dataset the proposed algorithm can accurately estimate the Cole-impedance parameters.

### The frequency distribution of the estimated parameters ${\widehat{a}}_{1}$*,*${\widehat{a}}_{2}$ and ${\widehat{b}}_{1}$

The parameters of the Cole-impedance model have different frequency ranges over which they dominate the overall impedance. For example, *R*_*∞*_ is the theoretical resistance at high (e.g. infinite) frequency, while *R*_1_ is equal to *R*_0_-*R*_*∞*_, where *R*_0_ is the theoretical resistance at low (e.g. *ω* = *0* rad/s) frequency. This results from the impedance of the constant phase element (*C*) which is very high at low frequency (resulting in the majority of the applied current for an impedance measurement to flow through *R*_1_), while it has very low impedance at high frequency (resulting in the majority of the current flowing through it and not *R*_1_). The contribution of *C* to the overall impedance is expected to be most recognizable around the characteristic frequency (inverse of the time constant (*R*_1_*C*)^1/α^). Therefore, it can be expected that elements of the arrays *a*_*1*_(*ω*_i_), *a*_*2*_(*ω*_i_) and *b*_*1*_(*ω*_i_) will also have different frequency distributions.

**Figure 4:**
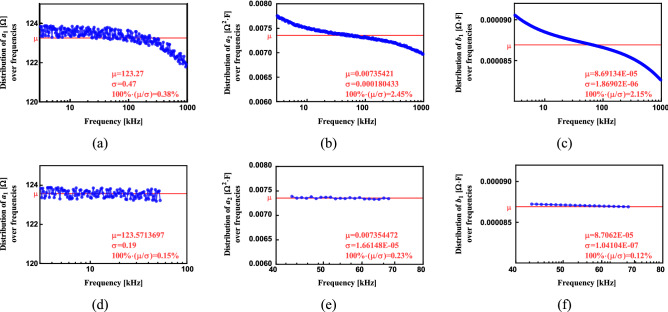
The frequency distribution of estimated values with Eqs. (10)–(12) in complete frequency range: (**a**) parameter *a*_1_, (**b**) parameter *a*_2_, (**c**) parameter *b*_1_; and in reduced frequency range: (**d**) parameter *a*_1_, (**e**) parameter *a*_2_, (**f**) parameter *b*_1_.

Our initial analysis of this effect utilizes the reference dataset (*R*_*∞*_ = 84.40 Ω, *R*_*1*_ = 39.20 Ω, *C* = 2.31 µF and *α* = 0.747)^19^ with 0.25% noise, 3 kHz–1 MHz frequency band, and *N* = 256 logarithmically spaced points. Estimated values of the Cole-model parameters using Method 1 applied to this dataset are: ${\widehat{R}}_{\infty }$=84.62 Ω, ${\widehat{R}}_{1}$=38.65 Ω, $\widehat{C}$=2.25 µF and $\widehat{\alpha }$=0.75. The frequency distributions of elements of arrays *a*_*1*_(*f*_i_), *a*_*2*_(*f*_i_) and *b*_*1*_(*f*_i_) are presented with blue dots in the Fig. 4 (a)-(c). These values are calculated at different frequencies but for the final estimated value of α = 0.75. The average value (µ) of all presented values is shown as a red solid line in each graph. The standard deviation (σ) and ratio of σ/µ in % are also indicated with text labels on those figures. As it can be seen in Fig. 4a, the deviation of estimated values of *a*_*1*_(*f*_i_) from the average (µ) is increasing at higher frequencies. Observing the deviations of estimated values of *a*_*2*_(*f*_i_) and *b*_*1*_(*f*_i_) in Fig. 4b and c, the deviations are the smallest in the central frequency band; approximately 30–100 kHz for *a*_*2*_(*f*_i_) and 60–100 kHz for *b*_*1*_(*f*_i_).

In the next step only lower frequencies (from *i* = 1 to *N*/2–1) are used for *a*_*1*_(*f*_i_) estimation and central frequencies (from i = *N*/2–10 to *N*/2 + 10) for *a*_*2*_(*f*_i_) and *b*_*1*_(*f*_i_). These specific frequency ranges are chosen based on the subjective analysis of results shown in Fig. 4a–c. Further analysis is needed for a generalized approach to the selection of frequency-points, however the bands selected here do illustrate how reducing the frequency band to subsets where the behavior is dominated by that parameter improves its estimation. To illustrate, estimated values of model parameters using these reduced frequency bands are: ${\widehat{R}}_{\infty }$=84.47 Ω, ${\widehat{R}}_{1}$=39.10 Ω, $\widehat{C}$=2.23 µF and $\widehat{\alpha }$=0.75. Note this improves the estimation accuracy with reduction of relative errors from 0.255% to 0.088%, and from 1.412% to 0.26% for ${\widehat{R}}_{\infty }$ and ${\widehat{R}}_{1}$, respectively. The frequency distributions of elements of arrays *a*_*1*_(*f*_i_), *a*_*2*_(*f*_i_) and *b*_*1*_(*f*_i_) from the reduced frequency estimation process are presented with blue dots in the Fig. 4d–f. Ratios of σ/µ for *a*_*1*_(*f*_i_), *a*_*2*_(*f*_i_) and *b*_*1*_(*f*_i_) are decreased from 0.38%, 2.45% and 2.15% to 0.15%, 0.23% and 0.12% respectively, indicating much more stable estimations. Therefore, there is a possibility that the complete set of measured frequencies are not needed to estimate all parameters which can reduce the computational cost of the algorithm. But further development of this approach is required to develop approaches to identify the optimum frequency band based on the collected measurements (which is planned for future works).

### Estimation with reduced datapoints

**Figure 5:**
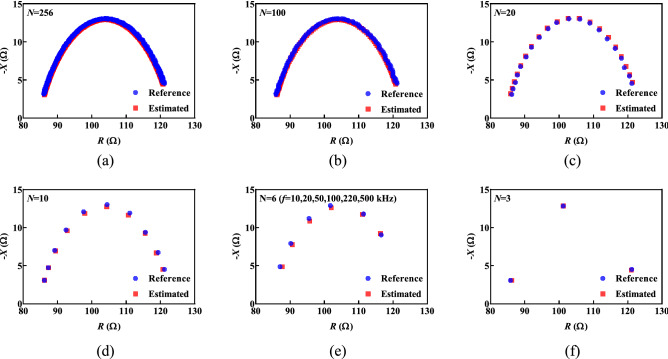
The Nyquist plots of reference and estimated values for different number of measurement points: (**a**) 256, (**b**) 100, (**c**) 20, (**d**) 10, (**e**) 6 and (**f**) 3.

The parameter estimation with the proposed algorithm was analyzed using datasets with a frequency range from 3 kHz to 1 MHz with 256 logarithmically spaced points to facilitate direct comparison with reference^19^. However, reducing the number of frequency points in dataset may improve the performance of portable/wearable devices with constrained resources in terms of both memory and power. To evaluate the effect of reducing datapoints on the algorithm performance, the proposed estimation method was applied to the reference dataset (*R*_*∞*_ = 84.40 Ω, *R*_*1*_ = 39.20 Ω, *C* = 2.31 µF and *α* = 0.747)^19^ with 0.25% noise, while number of points initially was *N* = 256, and then was decreased to 100, 20, 6, and 3. The estimated parameters for each case were: *N* = 256 (${\widehat{R}}_{\infty }$=84.63 Ω, ${\widehat{R}}_{1}$=38.65 Ω, $\widehat{C}$=2.25 µF, $\widehat{\alpha }$=0.75), *N* = 100 (${\widehat{R}}_{\infty }$=84.63 Ω, ${\widehat{R}}_{1}$=38.65 Ω, $\widehat{C}$=2.25 µF, $\widehat{\alpha }$=0.75), *N* = 20 (${\widehat{R}}_{\infty }$=84.15 Ω, ${\widehat{R}}_{1}$=39.99 Ω, $\widehat{C}$=2.49 µF, $\widehat{\alpha }$=0.74), *N* = 10 (${\widehat{R}}_{\infty }$=84.58 Ω, ${\widehat{R}}_{1}$=38.81 Ω, $\widehat{C}$=2.54 µF, $\widehat{\alpha }$=0.74), *N* = 6 (${\widehat{R}}_{\infty }$=84.80 Ω, ${\widehat{R}}_{1}$=39.50 Ω, $\widehat{C}$=2.94 µF $, \widehat{\alpha }$=0.73), and *N* = 3 (${\widehat{R}}_{\infty }$=84.56 Ω, ${\widehat{R}}_{1}$=39.03 Ω, $\widehat{C}$=2.21 µF , $\widehat{\alpha }$=0.75). The *N* = 3 case is presented because it represents the theoretical minimum for the parameter estimation because three points are needed using the central difference approximation of the first derivative. Frequency range from 10 to 500 kHz was used for *N* = 6^30^. The Nyquist plots of the reference and estimated impedance for each value of *N* are shown in Fig. 5a–f. Each case shows very good agreement with the ideal reference values confirming that reducing datapoints (even down to the theoretical limit) did not significantly impact the algorithm accuracy.

### Embedded hardware-based estimations

The noisy datasets used in subsection "Comparison with reference" ^19^ to test the proposed algorithm on a laptop computer were also used to test the algorithm on three embedded hardware platforms. The reference values of resistance and reactance were stored as arrays in the flash memory of the microcontrollers. Identical program code was compiled for each of the three platforms (Arduino Mega2560, Raspberry Pi Pico and XIAO SAMD21) using the Arduino IDE. After successful compiling and programming, the algorithm was executed on each platform. The comparison of estimated parameter values using our method deployed on the laptop, our method deployed on the embedded hardware platforms, and the CNLS-based method from^19^ deployed on laptop and Raspberry Pi 3 board are given in Table 3. The proposed method was optimized for execution on the embedded hardware by altering the brute-force search/step of α (originally proposed as a search from 0 to 1 in steps of 0.01). Here, a course search was performed with an α-step of 0.1 (from 0.1 to 0.9) with a second fine search performed with an α-step of 0.01. The fine search was performed using the course search value with the lowest error (α_1_) for the precise search in the range from α_1_ − 0.1 to α_1_ + 0.1. This approach slightly increases the utilized flash and RAM resources, but reduces the number of iterations to find optimal value of α. Consequently, the consumed energy is reduced which is a very important consideration for portable and wearable devices. For example, with a fixed step of 0.01 to sweep the range from 0 to 1 requires 101 iterations. But if the two-step estimation is performed, then 11 iterations are needed for the 0.1 step course sweep and 21 steps for the precise sweep (32 iterations total). Therefore, total execution time and energy consumption are reduced by approximately one-third.

**Table 3:** Comparison of estimated values of model parameters using different hardware platforms.

	*R*_*∞*_ (Ω)	*R*_*1*_ (Ω)	*C* (µF)	α	*t*_*exe*_ (s)
Reference	84.40	39.20	2.31	0.747	NA∙
CNLS^19^, (MATLAB 2016a on Dell Precession 3240)	84.56	38.65	2.01	0.759	14.76
CNLS^19^, (Python 3 on Raspberry Pi 3)	84.47	38.98	2.21	0.751	31.18
Our method (MATLAB 2013b on Dell G5)	84.62	38.65	2.25	0.75	0.15
Our method (Arduino Mega2560)	84.62	38.65	2.25	0.75	22.11
Our method (Raspberry Pi Pico)	84.62	38.65	2.25	0.75	10.05
Our method (XIAO SAMD21)	84.62	38.65	2.25	0.75	124.67

*NA* Not applicable.

From the results provided in Table 3, our method deployed on the Raspberry Pi Pico (operating on clock speed of 133 MHz) required slightly higher *t*_*exe*_ than the more powerful Raspberry Pi 3 board with clock speed of 1.2 GHz^31^. This highlights the benefit of using the proposed algorithm which did not require Python (an interpreted language) and supporting libraries. Note that the estimation accuracy is similar for all analyzed methods, confirming that the proposed algorithm has similar performance to established methods.

For each of the three tested embedded platforms, the default configuration was used during program execution. This approach was adopted so that results represented performance for users using the platforms “as it is” without specialized configuration. We hypothesize that further improvements in the algorithm performance can be achieved by optimizing the configuration of the each platform, but future research is needed to confirm.

An interesting observation is that the compiled programs for each of the embedded platforms required different program code size and SRAM. This is expected because of the different microcontroller families and architectures (8-bits and 32-bits). Almost 66 kB of flash was required for the deployment on the Raspberry Pi Pico, but only 55kB and 16 kB of flash were required for the XIAO SAMD21 and Arduino Mega2560, respectively. Deployment on the Raspberry Pi Pico needed a lower percentage of available RAM (12%) when compared to 67% for the Arduino Mega2560. By default, the compiler does not provide usage of RAM in case of XIAO boards preventing its comparison here.

Because current draw and power consumption are important considerations for portable/wearable systems (with aims to reduce hardware size and increase battery life) the current/energy consumption of each hardware platform executing the proposed algorithm was measured. The current consumption of each embedded platform was obtained using a Siglent SPD3303C DC power supply and Sanwa CD770 multimeter. The Siglent SPD3303C was set to output 5 V and the Sanwa CD770 was connected in series (as an ammeter) with the SPD3303C and embedded platform. The current consumption was consistent across program execution and estimation of the parameters. The XIAO SAMD21 had the lowest input current (13.29 mA), followed by the Raspberry Pi Pico (22.18 mA) and Arduino Mega2560 (53.3 mA). Using the current measurements, the energy consumption to perform the estimation (obtained by multiplication of the measured current consumption and estimation time given in Table 3) was 1.11 J for the Raspberry Pico. 5.97 J for the Arduino Mega2560, and 8.73 J for the XIAO SAMD21.

## Conclusion

In this work, we proposed a novel method for estimation of the Cole-impedance model parameters from bioimpedance datasets that does not use complex least squares methods. The proposed method showed a very good estimation accuracy, with improvements in terms of estimation speed when compared to related work from the literature. Further, increases in dataset noise and reductions in the number of datapoints in a dataset did not significantly degrade the accuracy of the algorithm.

This method is developed to support portable/wearable bioimpedance instruments with on-board processing requirements and limited computational hardware. As such, the performance of the algorithm was evaluated on three embedded platforms, the Arduino Mega2560, Raspberry Pi Pico and XIAO SAMD21. All platforms were shown capable of executing the proposed algorithm with accuracy comparable to laptop-based implementations (validating the algorithm for use on embedded hardware). Overall, the Raspberry Pi Pico was the platform with both the fastest execution time and smallest energy consumption.

Overall, the novelty and contributions of our work are: (1) a method for parameter estimation of the Cole-bioimpedance model suitable for deployment on low-complexity units (such as 8-bits microcontrollers), and (2) performance comparison of three embedded hardware-based platforms for identification of an appropriate system for future portable bioimpedance systems using the proposed algorithm.

Our future work will be directed towards the development of an integrated system for bioimpedance measurements and *in-situ* Cole-model parameter estimation. Moreover, we plan to use the presented method with experimentally obtained bioimpedance to resolve a limitation of the current work. Bioimpedance data collected from real tissues (and not synthetically generated datasets) is influenced by factors including electrode impedance (impacting low frequency measurements) and parasitic capacitances (impacting high frequency measurements). These factors can degrade the "fit" of the impedance data to the Cole-impedance model and is expected to degrade performance of the proposed algorithm. To resolve, future methods that identify features of data degradation and either correct or remove (prior to estimation of the Cole-impedance parameters) are needed. Further efforts will also investigation how to develop algorithms to fit more generalized and complex models beyond the Cole-impedance model to increase the possible range of bioimpedance datasets to which it can be applied.

## Data Availability

The datasets generated during and/or analyzed during the current study are available from the corresponding author on reasonable request.
